# Prognostic Role of Phospho-STAT3 in Patients with Cancers of the Digestive System: A Systematic Review and Meta-Analysis

**DOI:** 10.1371/journal.pone.0127356

**Published:** 2015-05-29

**Authors:** Mu-xing Li, Xin-yu Bi, Zhen Huang, Jian-jun Zhao, Yue Han, Zhi-yu Li, Ye-fan Zhang, Yuan Li, Xiao Chen, Xu-hui Hu, Hong Zhao, Jian-qiang Cai

**Affiliations:** 1 Department of Abdominal Surgical Oncology, Cancer Hospital, Chinese Academy of Medical Sciences and Peking Union Medical College (CAMS & PUMC), NO.17, Panjiayuan Nanli, Beijing, 100021, P. R. China; 2 Department of Radiofrequency Ablation, Cancer Hospital, Chinese Academy of Medical Sciences and Peking Union Medical College (CAMS & PUMC), NO.17, Panjiayuan Nanli, Beijing, 100021, P. R. China; Fondazione IRCCS Istituto Nazionale dei Tumori, ITALY

## Abstract

**Objective:**

The definite prognostic role of p-STAT3 has not been well defined. We performed a meta-analysis evaluating the prognostic role of p-STAT3 expression in patients with digestive system cancers.

**Methods:**

We searched the available articles reporting the prognostic value of p-STAT3 in patients with cancers of the digestive system, mainly including colorectal cancer, gastric cancer, hepatocellular carcinoma, esophagus cancer and pancreatic cancer. The pooled hazard ratios (HRs) with 95 % confidence intervals (95 % CIs) of overall survival (OS) and disease-free survival (DFS) were used to assess the prognostic role of p-STAT3 expression level in cancer tissues. And the association between p-STAT3 expression and clinicopathological characteristics was evaluated.

**Results:**

A total of 22 studies with 3585 patients were finally enrolled in the meta-analysis. The results showed that elevated p-STAT3 expression level predicted inferior OS (HR=1.809, 95% CI: 1.442-2.270, P<0.001) and DFS (HR=1.481, 95% CI: 1.028-2.133, P= 0.035) in patients with malignant cancers of the digestive system. Increased expression of p-STAT3 is significantly related with tumor cell differentiation (Odds ratio (OR) =1.895, 95% CI: 1.364-2.632, P<0.001) and lymph node metastases (OR=2.108, 95% CI: 1.104-4.024, P=0.024). Sensitivity analysis suggested that the pooled HR was stable and omitting a single study did not change the significance of the pooled HR. Funnel plots and Egger’s tests revealed there was no significant publication bias in the meta-analysis.

**Conclusion:**

Phospho-STAT3 might be a prognostic factor of patients with digestive system cancers. More well designed studies with adequate follow-up are needed to gain a thorough understanding of the prognostic role of p-STAT3.

## Introduction

Cancers derived from the digestive system, mainly including esophagus cancer, colorectal cancer, gastric cancer, hepatocellular carcinoma, pancreatic cancer, et al., account for a majority portion of the most killing malignant cancers worldwide [1, 2]. Digestive system cancers are featured by the aggressive biological behavior and unfavorable clinical outcome [3]. Despite the improvement of diagnostic and therapeutic approaches in the past decades, the prognosis of digestive system cancers remains to be dismal mainly due to local recurrence and distal metastases [1]. Currently, the designation of treatment strategy mainly depends on the TNM stage of tumor. It is common to observe that patients at the same TNM stage may have various clinical outcomes [4]. Molecular based prognostic factors could act as an implement of the current staging system. Thus, it is essential to identify molecular prognostic factors of digestive system cancers which aids in rational stratification of the patients according to the clinical prognosis as well as provides us with the potential therapeutic targets.

Signal transducer and activator of transcription proteins (STATs) are often activated through tyrosine phosphorylation and are then converted into its active form as phosphorylated STATs (p-STATs) [5]. Among the STATs family members, STAT3 is a prominent molecular hub connecting multiple vital molecular pathways involved in cancer progression. p-STAT3 can trigger gene expression by interacting with STAT cognate sequence in the DNA or numerous regulatory proteins [6], which further modulate the cellular biological behaviors including the cellular cycle [7], epithelial-mesenchymal transition (EMT) [8], the inflammatory responses [9] and angiogenesis [10].

Numerous studies indicated that alternation of p-STAT3 expression in tumor samples was associated with prognosis of various human malignancies such as breast cancer [11], lung cancer [12], lymphoma [13]. However, the exact prognostic role of p-STAT3 in cancers with the digestive systems remains to be unsettled. Several studies showed that p-STAT3 overexpression could significantly predict unfavorable outcome in patients with colorectal cancer [14], gastric cancer [15], hepatocellular carcinoma [16], esophagus cancer [17] and pancreatic cancer [18]; whereas the study by Monnien et al. [19] revealed that elevated expression of p-STAT3 was significantly related with advantageous clinical prognosis in colorectal cancer patients; and some other studies unveiled no significant association between p-STAT3 expression and the survival outcome in patients with gastric cancer [20], hepatocellular carcinoma [21], esophagus cancer [22] and pancreatic cancer [23]. Therefore we searched the available articles and performed the present meta-analysis in order to explore the prognostic value of p-STAT3 in patients with digestive system cancers. Besides, the association between p-STAT3 expression and the clinicopathological characteristics was assessed.

## Materials and Methods

### Search strategy

Literature search of MEDLINE, Web of Science, Cochrane library, EMBASE from their inception to September, 2014 was carefully performed. The following retrieval strategy was used: (‘cancer’ OR ‘tumor’ OR ‘tumour’ OR ‘neoplasm’ OR ‘carcinoma’ OR ‘adenocarcinoma’) AND (‘phosphorylated signal transducer and activator of transcription3’ OR ‘phosphate STAT3’ OR ‘phospho-STAT3’ OR ‘p-STAT3’) AND (‘prognosis’ OR ‘prognostic’ OR ‘outcome’). The reference list of each manuscript was manually screened in order to gain the potential related articles. No advanced limitations were additionally set. The written language was limited to English. Only articles published in peer-review journals were admitted into our further analysis.

### Study inclusion/exclusion criteria

Li MX and Bi XY independently scrutinized the initially identified articles. Studies were considered eligible if the following criteria were fulfilled: (1) they studied patients with digestive system cancers (i.e. gastric cancer, hepatocellular carcinoma, colorectal cancer, esophagus cancer and pancreatic cancer). (2) Expressions of p-STAT3 were measured in the tumor tissue samples. (3) studies presenting data regarding association between p-STAT3 expression and survival outcome or clinicopathological information such as tumor differentiation, TNM stages, and lymph node metastasis; (4) HRs (ORs) and 95% CI were directly extracted or synthesized by the relevant published data [24]. And HRs and 95% CI in terms of the survival outcome should be produced by the multivariate analysis; (5) for articles with duplicated or overlapping study population; only the most complete ones were enrolled. Agreement was reached by discussion.

The following items were defined as the exclusion criteria: (1) literature published as abstracts, letters, editorials, expert opinions, reviews and case reports; (2) articles without sufficient data to obtain the HRs (ORs) and CI; (3) researches based on cancer cells or animal models but not based on patients.

### Data extraction

The following data of each article was extracted: (1) general information including first author, publication year, country (area) of origin, age and gender of the study patients, sample size, and the follow-up duration; (2) clinicopathological characteristics including TNM stage, differential grade and lymph node metastasis; (3) HRs and 95% CIs produced by multivariate analysis investigating the relationship between elevated level of p-STAT3 and OS or DFS; (4) antibodies used for IHC; (5) scoring method and cut-off value defining “elevated expression” of p-STAT3. The index of scoring method in immunohistochemical staining (IHC) mainly included staining extent (E), intensity (I) and staining extent& intensity (EI). As the cut-off score for positive expression of p-STAT3 was not uniform among the studies, positive p-STAT3 expression was defined with respect to the original contents. Since half of the enrolled studies (11/22) used rabbit polyclonal antibody as the primary antibody in IHC, primary antibodies were classified as rabbit polyclonal antibody and other antibodies. Tumor cell differentiation grade was dichotomized as well/moderate and poor differentiation. TNM stage was subdivided as I/II and III/IV. Status of lymph node metastasis (N-status) was categorized as positive lymph node metastasis and negative lymph node metastasis.

### Quality assessment

Two authors (Li MX and Bi XY) independently conducted the quality assessment with the Newcastle–Ottawa Quality Assessment Scale (NOS) [25]. The assessment of the scale mainly includes selection of cases, comparability of populations, and ascertainment of exposure to risks. NOS scores of ≥ 6 were assigned as high-quality studies. Consensus was reached by discussion when disparity occurred.

### Statistical analysis

For each meta-analysis, the Cochrane’s Q statistic was undertaken to assess the heterogeneity of the trials. When combining the data, the random effect model or the fixed effect model was selected according the level of between study heterogeneity. A random effect model was used if severe between study heterogeneity was observed (I^2^≥50%); the fixed effect model was applied if there was no remarkable inter-study heterogeneity (I^2^<50%). All statistical tests were two-sided and P<0.05 was considered statistical significant. Publication bias was assessed by Begg’s funnel plot and Egger’s linear regression test [26]. All the analyses were conducted using STATA statistical software package version 12.0 (STATA Corp., College Station, Texas, USA).

## Results

### The description of the included studies

**Fig 1:**
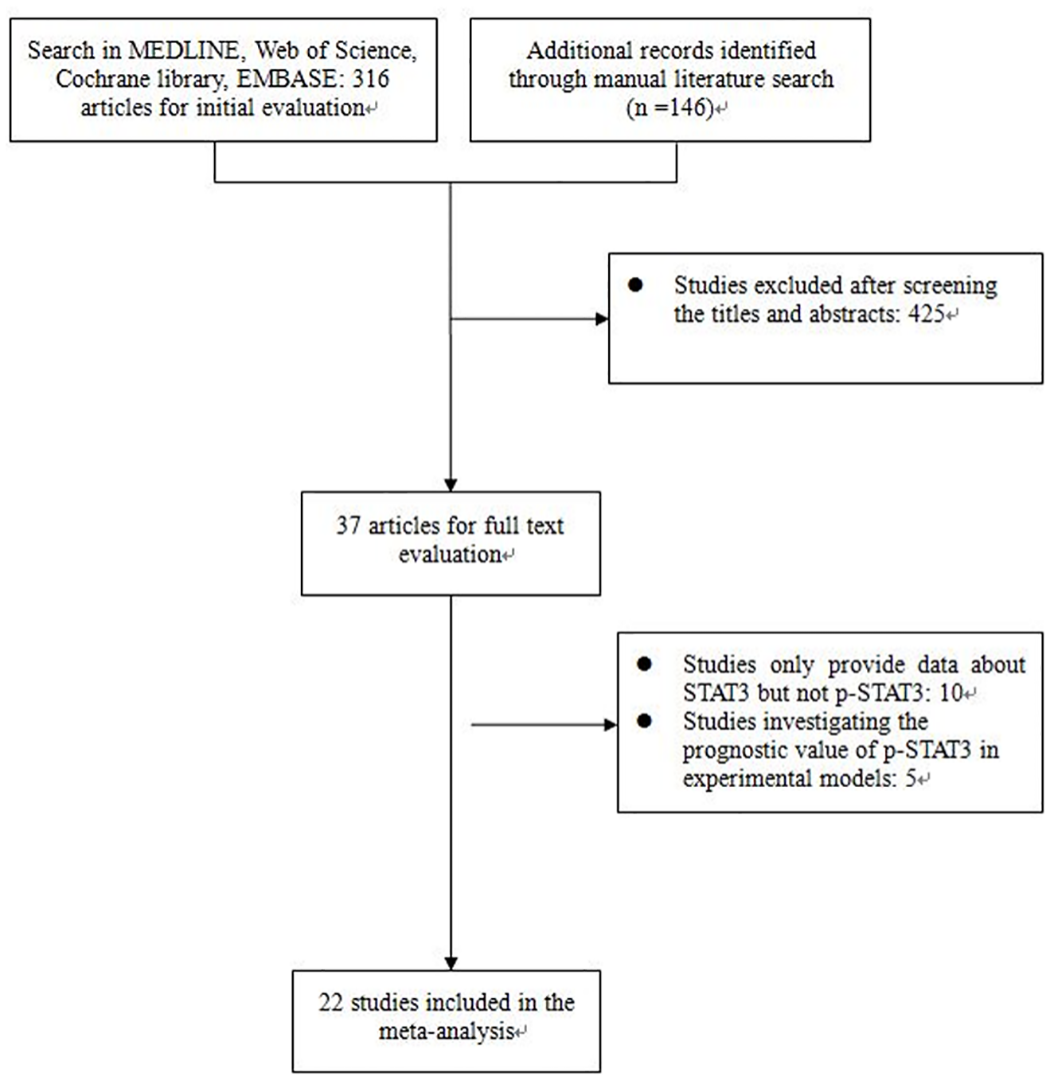
Flow chart for the selection of articles to include.

The initial search yielded a total of 462 articles. After meticulous inspection of the primary search results, 22 studies [14–23, 27–38] published between 2006 and 2014 were finally enrolled in our meta-analysis (details in Table 1). The flowchart of the selection of enrolled studies was illustrated in Fig 1. We found that Deng was the first author of two included articles [30, 33] which studied patients from the same institution but from separated time period. We marked them as Deng1 and Deng2 respectively.

**Table 1:** Main characteristics of all the studies included in the meta-analysis.

First author	Year	Study region	Primary site	Treatment	NO. (M/F)	Age	Follow-up(months)	N status(-/+)
Kusaba [27]	2006	Japan	colorectal	surgery	66/42	65.6ys range:44–86ys	Median:43.7ms	74/34
							Range:0.71–60ms	
Monnien [19]	2010	France	colorectal	surgery	76/28	Median: 66ys	>60ms	67/31
Morikawa [28]	2011	USA	colorectal	surgery	266/458	≥65ys:454, <65ys:270	Median: 129ms	NR
Dobi [14]	2012	France	colorectal	chemotherapy	50/44	≤70ys:60, >70ys:34	>120ms	NR
Yakata [29]	2006	Japan	gastric	surgery	63/48	Median:68.9ys	NR	79/32
						Range:38–89ys		
Lee [15]	2009	Korea	gastric	surgery	210/101	Median: 53ys	>60ms	NR
Deng [30]	2010	China Mainland	gastric	surgery	37/16	Median:55ys	Median:35ms	NR
						range:31–78ys	Range:4–85ms	
Inokuchi [20]	2011	Japan	gastric	surgery	88/38	≥70ys:41, <70ys:85	73ms	59/76
Woo [31]	2011	Korea	gastric	surgery	193/92	NR	Mean:51ms	102/183
Xiong [32]	2012	China Mainland	gastric	NR	176/86	Mean 59.3ys	Mean: 39.7ms	NR
Deng [33]	2013	China Mainland	gastric	surgery	76/38	Median:56.7ys	Median:38ms	42/68
						Range:29–83ys	Range:2–108ms	
Song [34]	2014	China Mainland	gastric	surgery	46/14	Median:60ys	Range:6–72ms	0/60
Wu [35]	2014	China Mainland	gastric	surgery	43/17	Mean:62ys	Median:23ms	NR
						Range:37–90ys	Range:1–79ms	
Zhang [16]	2012	Austria	hepatocellular	surgery	80/20	Mean:55.1ys	NR	NR
						Range:28–77ys		
Wu [36]	2011	China Mainland	hepatocellular	surgery	115/23	≤55ys:86,>55ys:52	NR	NR
Mano [21]	2013	Japan	hepatocellular	surgery	81/20	positive 63.9±7.3ys	Median:1391days	NR
						negative 63.6±9.5ys	Range:36–3289days	
You [17]	2011	China Mainland	esophagus	surgery	68/32	62.2±6.6ys	Median:16.8ms	NR
							Range:0.8–69.2ms	
Schoppmann [37]	2012	Austria	esophagus	surgery	253/72	63±10ys	52ms	NR
Chen [22]	2013	Taiwan	esophagus	radiotherapy	173	NR	NR	48/125
Denley [18]	2013	UK	pancreatic	surgery	43/43	Median:64ys	NR	NR
						Range:38–77ys		
Huang [23]	2013	China Mainland	pancreatic	surgery	50/21	Median:67ys	Mean:15.9ms	NR
						Range:40–80ys	Range:1–101ms	
Koperek [38]	2013	Caucasian	pancreatic	surgery	48/31	66±11ys	629±594days	NR
Study Cohort	Tumor differentiation (well or moderate/poor)	TNM(I+II/III+IV)	Antibody	Scoring Method	Cut-off	NO.of positive expression (%)	Survival analysis	HR
Kusaba [27]	100/4	NR	goat polyclonal antibody	E	15%	62(57.4%)	NR	R(M)
Monnien [19]	97/1	0/104	rabbit polyclonal antibody	E	15%	39(37.5%)	OS	R(M)
Morikawa [28]	NR	384/301	rabbit polyclonal antibody	I	NR	131(18%,)	OS	R(M)
Dobi [14]	362/0	19/75	rabbit polyclonal antibody	E	15%	23(24.5%)	OS,DFS	R(M)
Yakata [29]	NR	NR	goat polyclonal antibody	E	10%	55(49.5%)	NR	R(M)
Lee [15]	101/166	144/167	rabbit polyclonal antibody	E	1%	79(26.1%)	OS,DFS	R(M)
Deng [30]	NR	NR	rabbit polyclonal antibody	E	10%	26(49.1%)	OS	R(M)
Inokuchi [20]	NR	NR	rabbit polyclonal antibody	E	10%	52(41%)	OS	R(M)
Woo [31]	NR	NR	NS	E	1%	103(36%)	NR	R(M)
Xiong [32]	80/182 1+2/3+4	93/169	NS	E	15%	136(51.9%)	OS	R(M)
Deng [33]	NR	NR	rabbit polyclonal antibody	EI	25%	89(78.07%)	OS	E(M)
Song [34]	26/34 1+2/3	26/34	rabbit polyclonal antibody	EI	4	35(58.3%)	OS	R(M)
Wu [35]	NR	NR	NS	E	5	26(43.3%)	NR	R(M)
Zhang [16]	68/32 1+2/3+4	60/40	mouse monoclonal antibody	E	15%	58(58%)	OS	R(M)
Wu [36]	NR	106/31	NS	E	25%	75(54.3%)	NR	R(M)
Mano [21]	66/35 1+2/3	NR	rabbit monoclonal antibody	E	10.7%	36(36%)	OS,DFS	R(M)
You [17]	73/27	20/80	rabbit polyclonal antibody	EI	2	76(76%)	OS	R(M)
Schoppmann [37]	183/141	NR	rabbit monoclonal antibody	EI	10	144(44.4%)	OS,DFS	R(M)
Chen [22]	NR	45/128	NS	EI	2	82(47.4%)	OS,DFS	R(M)
Denley [18]	NR	NR	rabbit polyclonal antibody	EI	NR	29(33.7%)	OS	R(M)
Huang [23]	59/10	66/5	rabbit polyclonal antibody	E	5%	39(54.9%)	OS	R(M)
Koperek [38]	53/26	NR	rabbit monoclonal antibody	E	5%	33 (41.8%)	NR	R(M)

ms: months; ys: years; OS: overall survival; DFS: progression-free survival; HR: hazard ratio, obtained by reporting in text (R) or estimating (E). ‘‘M” means the HR come from multivariate analysis; E: extent; I: intensity; NR: not reported; NS: non-specific; Rabbit pAb: rabbit polyclonal antibody.

The sample sizes of the included cohorts ranged from 53 to 724. Colorectal cancer, gastric cancer, hepatocellular carcinoma, esophagus cancer and pancreatic cancer were studied in 4, 9, 3, 3 and 3 articles, respectively. All of these studies evaluated the expression of p-STAT3 in tumor samples by IHC. Different antibodies including rabbit polyclonal antibody (11 studies), rabbit monoclonal antibody (3 studies), mouse monoclonal antibody (1 study), and non-specific antibody (5 studies), goat polyclonal antibody (2 studies) were used. Study patients in 20 of the 22 included studies received surgical operation as the main treatment. Quality assessment revealed that only 3 [31, 36, 38]of the 22 studies gained a NOS<6 (details in Table 2).

**Table 2:** Quality assessment of eligible studies with Newcastle-Ottawa Scale.

Author	Year	Selection	Comparability	Outcome	NOS
Kusaba [27]	2006	★★*	★★*	★★*	6
Monnien [19]	2010	★★★*	★★*	★★*	7
Morikawa [28]	2011	★★★*	★★*	★★★*	8
Dobi [14]	2012	★★★*	★*	★★*	6
Yakata [29]	2006	★★★*	★*	★★*	6
Lee [15]	2009	★★*	★★*	★★*	6
Deng [30]	2010	★★^Δ^	★★*	★★*	6
Inokuchi [20]	2011	★★*	★★*	★★*	6
Woo [31]	2011	★★*	★*	★★*	5
Xiong [32]	2012	★★*	★★*	★★*	6
Deng [33]	2013	★★*	★★*	★★*	6
Song [34]	2014	★★*	★★*	★★*	6
Wu [35]	2014	★★★*	★*	★★*	6
Zhang [16]	2012	★★*	★★*	★★*	6
Wu [36]	2011	★★*	★*	★★^Δ^	5
Mano [21]	2013	★★*	★★*	★★*	6
You [17]	2011	★★*	★★*	★★*	6
Schoppmann [37]	2012	★★*	★★^Δ^	★★*	6
Chen [22]	2013	★★*	★★*	★★*	6
Denley [18]	2013	★★*	★★*	★★*	6
Huang [23]	2013	★★*	★★*	★★*	6
Koperek [38]	2013	★★*	★*	★★^Δ^	5

The table presented the final quality assessment score of the enrolled studies by the authors.

* The score was consistent in the initially separate assessment by Li MX and Bi XY.

^Δ^ The score was produced by the joint discussion.

### p-STAT3 and OS

**Fig 2:**
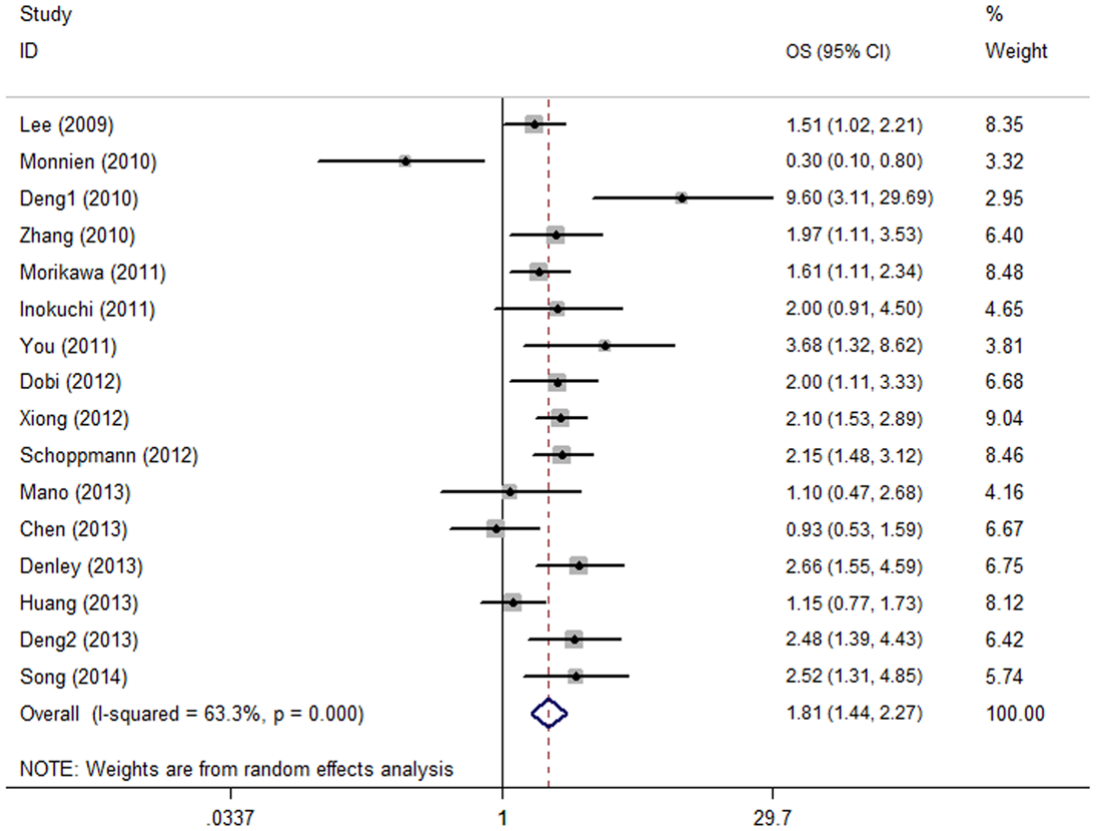
Forest plot of hazard ratio (HR) for the association between p-STAT3 overexpression and overall survival (OS) in patients with cancers of the digestive system with random effects model.

There were 16 cohorts presented the data of p-STAT3 expression and overall survival of the patients. Though with heterogeneity (I^2^ = 63.3%, P value of Q test for heterogeneity test (Ph) <0.001), pooled estimates showed that elevated p-STAT3 expression predicted poor OS with a pooling HR being 1.809 (95% CI: 1.442–2.270, P<0.001. Table 3, Fig 2).

**Table 3:** Meta-analysis results for OS and DFS.

Analysis	OS					DFS				
	N	HR(95% CI)	I^2^	Ph	P	N	HR(95% CI)	I^2^	Ph	P
	16	**1.809(1.442–2.270)**	63.3%	<0.001	**<0.001**	5	**1.481(1.028–2.133)**	71.4%	0.007	**0.035**
Subgroup1: digestive tract	12	**1.875(1.426–2.464)**	66.5%	0.001	**<0.001**					
Digestive gland	4	**1.636(1.056–2.536)**	57.4%	0.070	**0.028**					
Subgroup2: surgery	13	**1.871(1.425–2.458)**	65.0%	0.052	**<0.001**					
Non-surgical	2	1.361(0.640–2.895)	73.4%	0.001	0.423					
Subgroup3: Caucasian	5	**1.656(1.067–2.571)**	72.8%	0.005	**0.025**					
Asian	11	**1.873(1.415–2.481)**	61.7%	0.004	**<0.001**					
Subgroup4: E	8	**1.490(1.101–2.016)**	60.0%	0.015	**0.010**					
I	1	**1.610(1.109–2.338)**	-	-	**0.012**					
EI	7	**2.430(1.622–3.641)**	66.7%	0.006	**<0.001**					
Subgroup5: Sample size≥200	4	**1.846(1.542–2.209)**	0	0.416	**<0.001**					
Sample size<200	12	**1.818(1.276–2.591)**	70.9%	<0.001	**0.001**					
Subgroup6: Rabbit pAb	11	**1.918(1.394–2.640)**	69.0%	<0.001	**<0.001**					
others	3	**1.947(1.448–2.617)**	0	0.392	**<0.001**					

Ph: p value of Q test for heterogeneity test; N: number of studies (cohorts); HR: hazard ratio; 95% CI: 95% confidence interval; OS: overall survival; DFS: progression-free survival; E: extent; I: intensity; Rabbit pAb: rabbit polyclonal antibody

We stratified the pooled data by tumor site (digestive tract vs. digestive gland), main treatment (surgical vs. non-surgical), study region (Caucasian vs. Asian), scoring methods (E vs. I vs. EI), sample size (≥200 vs. <200) and the primary antibody (rabbit polyclonal antibody vs. others) used in IHC. Majority of the results of subgroup analyses were consistent with the overall result in the total study population (data shown in Table 3).

Of note, when performing the subgroup analyses stratified by sample size, we found that studies with sample size ≥ 200 gained an I^2^ as 0.0%; while the subgroup with sample size < 200 had an I^2^ as 70.9%. It suggested that sample size may explain the source of heterogeneity to some extent. We further performed the meta-regression analysis by tumor site, main treatment, study region, scoring methods, sample size and the primary antibody used in IHC. To our disappointment, we did not figure out a single factor that was responsible for the source of heterogeneity (data not shown).

### p-STAT3 and DFS

**Fig 3:**
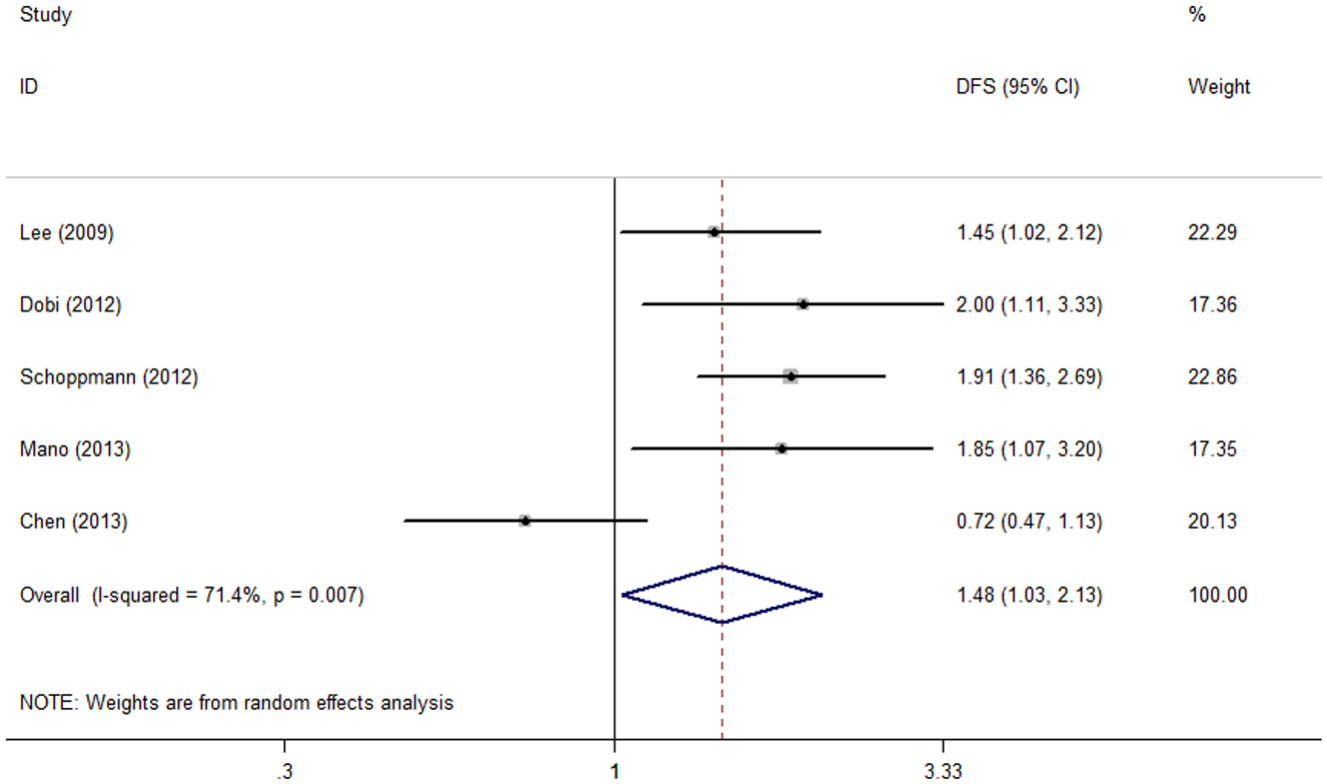
Forest plot of hazard ratio (HR) for the association between p-STAT3 overexpression and disease-free survival (DFS) in patients with cancers of the digestive system with random effects model.

Five cohorts reported the data concerning the association between p-STAT3 expression and disease-free survival of the enrolled patients. Meta-analysis adopting the random effect model revealed that elevated p-STAT3 expression was significantly associated with shorter DFS (HR = 1.481, 95% CI: 1.028–2.133, P = 0.035, Table 3, Fig 3) with observed heterogeneity (I^2^ = 71.4%, Ph = 0.007).

### p-STAT3 and clinical pathological factors

**Fig 4:**
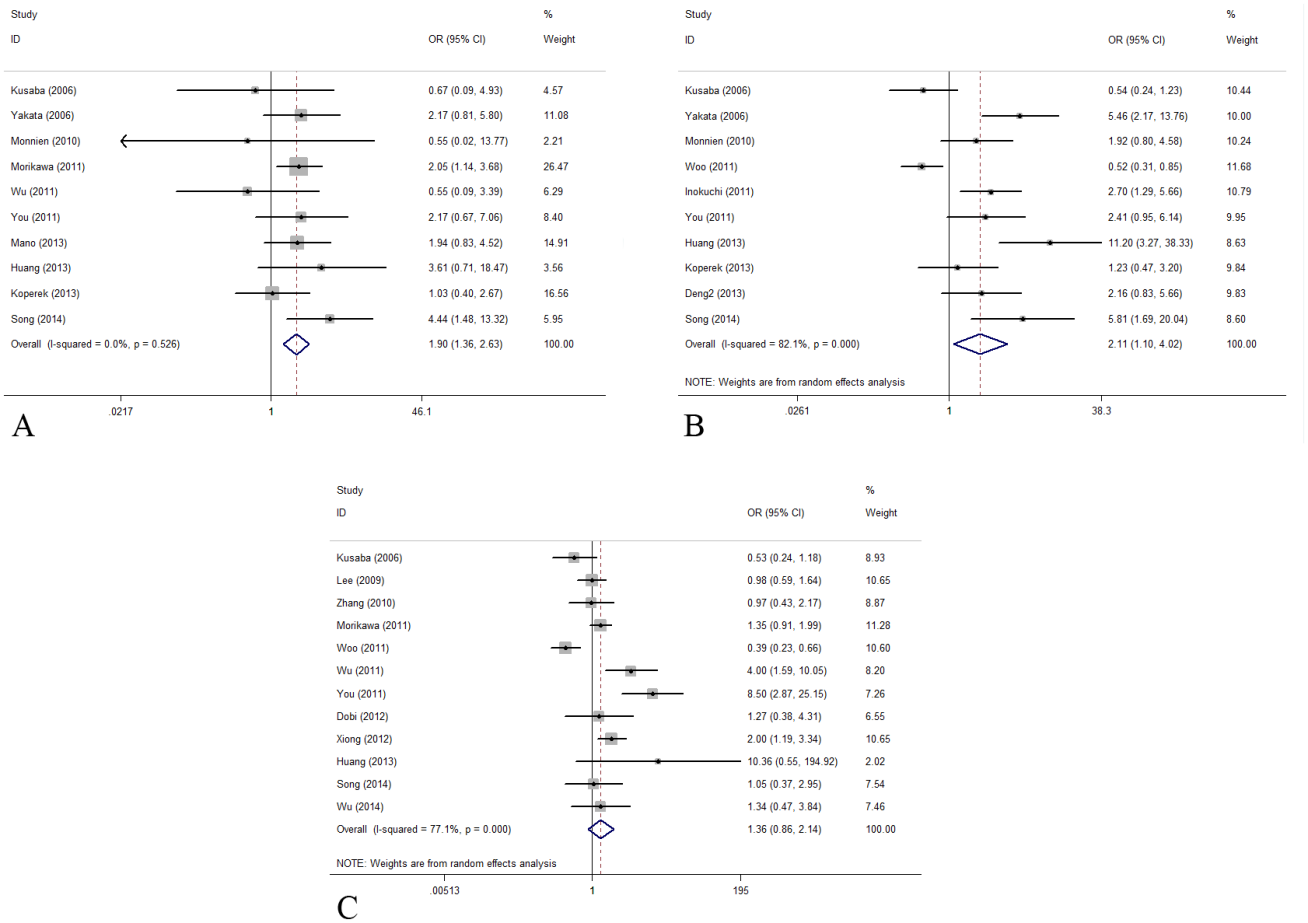
Forest plots of odds ratios (OR) for the association between p-STAT3 overexpression and clinicopathological features in digestive system cancer patients. (A) The relationship between p-STAT3 overexpression and tumor cell differentiation with fixed effects model (OR = 1.895, 95% CI: 1.364–2.632, P<0.001, I^2^ = 0, Ph = 0.526); (B) The relationship between p-STAT3 overexpression and lymph node metastases with random effects model (OR = 2.108, 95% CI: 1.104–4.024, P = 0.024, I^2^ = 82.1%, Ph<0.001); (C) OR for TNM stage with random effects model (OR = 1.355, 95% CI: 0.859–2.139, P = 0.192, I^2^ = 77.1%, Ph<0.001).

The secondary results of the present meta-analysis came as the relationship between p-STAT3 expression and the clinicopathological factors. Without observable heterogeneity, pooled estimates of 12 cohorts discovered that elevated p-STAT3 expression was significantly associated with poor tumor differentiation (OR = 1.895, 95% CI: 1.364–2.632, P<0.001, I^2^ = 0, Ph = 0.526, Fig 4A). Ten studies presented data about p-STAT3 expression and lymph node metastases, a combined OR of 2.108 revealed the positive relationship between increased p-STAT3 expression and positive N status (OR = 2.108, 95% CI: 1.104–4.024, P = 0.024, I^2^ = 82.1%, Ph<0.001, Fig 4B). In the meta-analysis assessing the association between p-STAT3 expression and TNM stage, the relationship failed to obtain the statistical significance (OR = 1.355, 95% CI: 0.859–2.139, P = 0.192, I^2^ = 77.1%, Ph<0.001, Fig 4C).

### Sensitivity analyses

To test the strength of our results, we removed an individual study each time and calculated the pooled HRs (or ORs) of the remaining studies. No significant differences were observed between the corresponding results and the overall results (data not shown), which indicated that our results were robust.

### Publication bias

**Fig 5:**
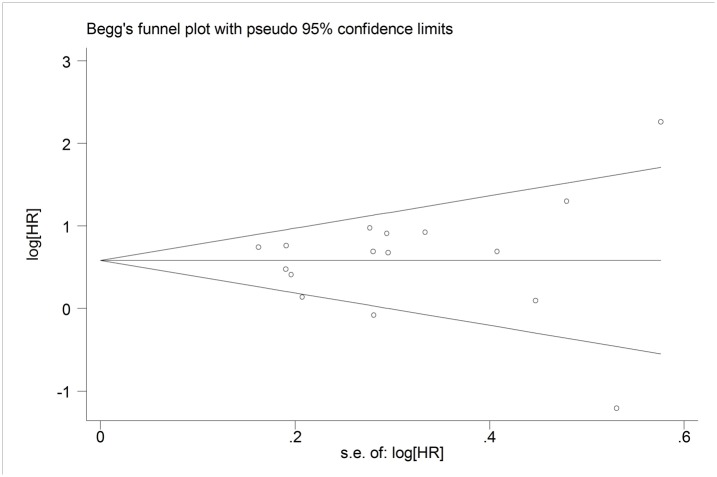
Funnel plot for p-STAT3 expression and overall survival (OS) in patients with cancers of the digestive system.

No obvious asymmetry was observed in the funnel plot of the meta-analysis evaluating the relationship between p-STAT3 expression and overall survival (Fig 5). And the P value of Egger’s test (P = 0.144) also indicated that there was no obvious publication bias.

## Discussion

To our knowledge, the present meta-analysis, involving a total 22 studies and 3585 patients, was the first meta-analysis systematically evaluating the prognostic value of p-STAT3 in patients with digestive system cancers. The results showed that elevated p-STAT3 expression level was a strong predictor of inferior OS and DFS in patients with malignant cancers of the digestive system. Majority of the results from subgroup analyses were similar with those from the overall study population, which indicated that the pooled results were robust. Additionally, increased p-STAT3 expression was also significantly interrelated with positive lymph node metastases status and poorer differentiation of tumor cells.

Among the tumor types evaluated, gastric cancer was the tumor type most linked with a worse outcome for patients who expressed high level of p-STAT3 (HR = 2.264, 95% CI: 1.629–3.147, P<0.001, I^2^ = 52.2%, S2 Table). As pooled estimates with limited number of enrolled studies are inclined to have insufficient statistical power, we dichotomized the enrolled studies as “digestive tract cancer” and “digestive gland cancer” in order to gain more statistical sound results. The respective results suggested that significant relationship between overexpression of p-STAT3 in tumor samples was detected both in the digestive tract cancer and digestive gland cancer subgroups.

The molecular functions of p-STAT3 in malignant tumors, mainly including its influence on cell cycle, inflammatory process and angiogenesis, have been extensively discussed in the recent years [39]. It has long been held that cytokines produced by inflammatory reaction contributed a lot in cancer progression. JAK/STAT3 pathway activation, which may be stimulated by the carcinogenetic inflammatory cytokine IL-6 through gp130, can lead to cell proliferation and antagonize cellular apoptosis [40]. In addition, EGFR activation can function through STAT3 phosphorylation. The activated downstream cytokines, such as TWIST, are able to fascinate the process of epithelial-mesenchymal transition (EMT), which is widely recognized as a critical step in lymph node/vascular metastasis [41]. Moreover, the interaction between VEGF expression and p-STAT3 plays an important role in the regulation of transcription of genes which are involved in angiogenesis [8]. These molecular mechanisms related with p-STAT3 may partially account for the molecular basis of reduced survival benefits and the unfavorable clinicopathological features.

Molecular targeted therapy has drawn cumulating attention theses years. Monoclonal antibodies or small molecule anti-tumor agents targeting the critical carcinogenic molecular hubs, such as VEGF, EGFR and HER-2, have earned appreciation in the anti-cancer treatment and have been adopted by the evidence based clinical guidelines. The growing number of preclinical studies in numerous malignances along with the clinical trials testing STAT3 inhibitors suggest that STAT3 (p-STAT3) remains a valid target for the treatment of malignant cancers [42]. Our results also suggest that molecular therapy countering p-STAT3 may shed light on the future development of molecular targeted therapy[43]. Several clinical trials assessing the therapeutic effects of STAT3 (p-STAT3) antagonists in patients with solid cancers (NCT01563302, NCT02058017, etc. http://www.clinicaltrials.gov/, Table 4) are underway. For cancers derived from the digestive system, a Phase I/Ib Study evaluating AZD9150 (a STAT3 inhibitor, ISIS-STAT3Rx) in Patients with Advanced/Metastatic Hepatocellular Carcinoma (NCT01839604, http://www.clinicaltrials.gov/, Table 4) is in process. We are looking forward that the results of the clinical trials will further unveil the value of p-STAT3 in oncological therapy.

**Table 4:** Ongoing studies evaluating STAT3 (p-STAT3) therapeutic strategies (from http://www.clinicaltrials.gov/).

Study	sponsor	Phase/setting	Experimental arm(s)
NCT01563302	Isis Pharmaceuticals	Phase 1/2, Open-label, Dose-escalation Study, Advanced Cancers	Three-hour IV infusions on Cycle 0 Days 1, 3, 5, and weekly three-hour IV infusions in Cycles 1 and beyond, on Days 1, 8, and 15 of each cycle.
NCT01568996	John Kirkwood	Phase 0, Atypical Nevi Melanoma	Low dose BSE-SFN:
			BSE-SFN will be orally administered at 50 μmol SFN for 28 days.
			Mid dose BSE-SFN:
			BSE-SFN will be orally administered at 100 μmol SFN for 28 days.
			High dose BSE-SFN:
			BSE-SFN will be orally administered at 200 μmol SFN for 28 days.
NCT02058017	National University Hospital, Singapore	Phase 1, Nasopharyngeal Carcinoma	Experimental: Part I & Part II
			Part I- This is a lead-in dose-finding, open-label, non-randomised arm of the study: Using a starting dose of 10mg per week, an accelerated dose titration escalation followed by a 3+3 design will be employed until MTD and recommended weekly dose are determined.
			Part II- This is a single-centre, open-label non-randomised phase II study evaluating OPB-51602 in stage III-IVB NPC conducted in the window period prior to definitive chemoradiotherapy. Eligible patients will receive OPB-51602 on a weekly basis (Day 1, 8, 15) at the recommended dose determined in part I for a total of 15 days prior to definitive chemoradiotherapy.
NCT01839604	AstraZeneca	Phase 1, Advanced Adult Hepatocellular Carcinoma, Hepatocellular Carcinoma Metastatic	Experimental: AZD9150, Intravenous infusion over 3 hours. There are two parts, dose escalation phase (Part A) and dose expansion phase (Part B).
NCT01066663	Dana-Farber Cancer Institute	Phase 1& Phase 2, Chronic Lymphocytic Leukemia, Small Lymphocytic Leukemia	Experimental: Pyrimethamine, Single daily oral 50 mg dose.

Furthermore, p-STAT3 expression in all of the enrolled articles was determined by IHC. IHC is a widely available method, but IHC is not strictly quantitative and there is no uniformly complied scoring system; therefore, interpretation of the staining results varies from person to person [44], which might potentially engender a certain degree of heterogeneity. Cut-off value defining elevated expression of p-STAT3 differed among the included cohorts: some authors arbitrarily defined it as 10%, 25% by staining extent or defined it as 2, 4, or 5 by the specific scoring system; in some studies, the cut-off value was determined as the mean/median value of the results by the specific scoring protocol in the respective article. Besides, diversities existed in the primary antibodies used in IHC, and the dilutions of the antibodies were not uniform, leading to a potential heterogeneity, because the sensitivity of the IHC may depend on the antibody concentration, fixation method and storage time [44–46]. The predicative value of elevated p-STAT3 expression in OS was not undermined by the subgroup analysis according to the primary antibody used. Of note, it was not possible to perform subgroup analysis stratified by all these technical issues, because limited number of studies shared the same laboratory procedure. Therefore a consistent and reproducible method to evaluate p-STAT3 expression is warranted.

Admittedly, there were some limitations in the present meta-analysis. First, the majority of the enrolled studies were retrospective. Thus some bias, such as selection bias, misclassification bias and information bias, may be present in the meta-analysis; Secondly, in order to gain more statistically robust data and sound accuracy of the pooled estimates, we only incorporated HRs and 95% CIs that were produced by the multivariate analysis in the present meta-analysis. We did not take studies that just present the Kaplan-Meier curves or studies that only present the HRs (95% CIs) from the univariate analysis. Therefore the number of the enrolled cohorts investigating the impaction of p-STAT3 expression on survival outcome is relatively limited. Thirdly, as the tumor samples used for IHC were commonly from the surgical resection, surgical operation remained to be the dominant treatment in majority of the enrolled population. It is possible that the results of our meta-analysis may have more hints to patients who underwent operation. Fourthly, though we did not detect obvious asymmetry in the funnel plots and evidence of publication bias in the Egger’s test, the combined outcomes may be relatively overestimated. Since small scale studies with negative results are prone to remain unpublished [26]. What is more, the subgroup analysis showed that smaller sample size may partially explain the source of heterogeneity. It could be explained by the fact that studies with smaller sample size are featured by the drawback of remarkable statistic instability. In the further meta-regression test, we did not find a single factor that was responsible for the sources of heterogeneity. We assumed that complex varieties in study design, study population, follow-up period, scoring system and laboratory protocol might contribute to the heterogeneity. In addition, we only searched limited online databases and only took English written articles into account. Though we tried our best to identify the relevant articles, the terms and algorithm adopted in the literature search may not be the best. The scope of the identified studies was somehow narrow.

In conclusion, the current meta-analysis suggests that p-STAT3 expression in the tumor sample of digestive system was a promising predictor of both OS and DFS. And level of p-STAT3 expression is closely related with lymph node metastasis and tumor cell differentiation. Future well designed studies with adequate follow up are needed. The promising therapeutic role of STAT3 (p-STAT3) targeted therapy will be further revealed by the ongoing clinical trials.

## Supporting Information

S1 Fig PRISMA Flow Diagram. (DOC)

S1 Table PRISMA Checklist. (DOC)

S2 Table Subgroup analysis stratified by tumor type evaluating the association between p-STAT3 overexpression and OS. (DOC)
