# Supplementary material for: Prognostic Role of Phospho-STAT3 in Patients with Cancers of the Digestive System: A Systematic Review and Meta-Analysis
Source: PLoS One. 2015 May 29;10(5):e0127356. doi: 10.1371/journal.pone.0127356 (PMC4449159; doi:10.1371/journal.pone.0127356)
Supplement: S1 Fig — (DOC) [file pone.0127356.s001.doc]

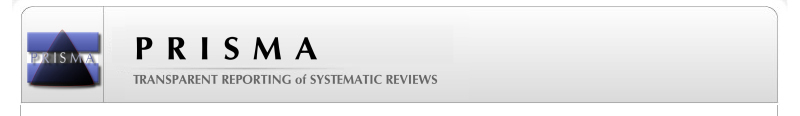
**PRISMA 2009 Flow Diagram**

**Screening**

**Included**

**Eligibility**

**Identification**

Search in MEDLINE, Web of Science, Cochrane library, EMBASE: 316 articles for initial evaluation

37 articles for full text evaluation

- Studies excluded after screening the titles and abstracts: 425
- Studies only provide data about STAT3 but not p-STAT3: 10
- Studies investigating the prognostic value of p-STAT3 in experimental models: 5

22 studies included in the meta-analysis

Additional records identified through manual literature search (n =146)
