# Supplementary material for: Prognostic Role of Phospho-STAT3 in Patients with Cancers of the Digestive System: A Systematic Review and Meta-Analysis
Source: PLoS One. 2015 May 29;10(5):e0127356. doi: 10.1371/journal.pone.0127356 (PMC4449159; doi:10.1371/journal.pone.0127356)
Supplement: S2 Table — (DOC) [file pone.0127356.s003.doc]

Table S3 Subgroup analysis stratified by tumor type evaluating the association between p-STAT3 overexpression and OS

| Analysis | OS | | | | |
| --- | --- | --- | --- | --- | --- |
|  | N | HR(95%CI) | I^2^ | Ph | P |
|  | 16 | **1.809(1.442-2.270)** | 63.3% | <0.001 | **<0.001** |
| Gastric cancer | 6 | **2.264(1.629-3.147)** | 52.2% | 0.063 | **<0.001** |
| Hepatocellular carcinoma | 2 | **1.654(1.020-2.682)** | 14.9% | 0.278 | **0.041** |
| Colorectal cancer | 3 | 1.149(0.523-2.526) | 80.8% | 0.006 | 0.729 |
| Esophagus cancer | 3 | 1.825(0.906-3.677) | 76.9% | 0.013 | 0.092 |
| Pancreatic cancer | 2 | 1.716(0.757-3.890) | 82.9% | 0.016 | 0.196 |

Ph: p value of Q test for heterogeneity test; N: number of studies (cohorts); HR: hazard ratio; 95% CI: 95% confidence interval;
